# Identification and Characterization of BMS-955176, a Second-Generation HIV-1 Maturation Inhibitor with Improved Potency, Antiviral Spectrum, and Gag Polymorphic Coverage

**DOI:** 10.1128/AAC.02560-15

**Published:** 2016-06-20

**Authors:** Beata Nowicka-Sans, Tricia Protack, Zeyu Lin, Zhufang Li, Sharon Zhang, Yongnian Sun, Himadri Samanta, Brian Terry, Zheng Liu, Yan Chen, Ny Sin, Sing-Yuen Sit, Jacob J. Swidorski, Jie Chen, Brian L. Venables, Matthew Healy, Nicholas A. Meanwell, Mark Cockett, Umesh Hanumegowda, Alicia Regueiro-Ren, Mark Krystal, Ira B. Dicker

**Affiliations:** a Bristol-Myers Squibb, Research and Development, Department of Virology, Wallingford, Connecticut, USA; b Bristol-Myers Squibb, Research and Development, Department of Discovery Chemistry, Wallingford, Connecticut, USA; c Bristol-Myers Squibb, Research and Development, Department of Genomics, Wallingford, Connecticut, USA; d Bristol-Myers Squibb, Research and Development, Department of Preclinical Optimization, Wallingford, Connecticut, USA

## Abstract

BMS-955176 is a second-generation human immunodeficiency virus type 1 (HIV-1) maturation inhibitor (MI). A first-generation MI, bevirimat, showed clinical efficacy in early-phase studies, but ∼50% of subjects had viruses with reduced susceptibility associated with naturally occurring polymorphisms in Gag near the site of MI action. MI potency was optimized using a panel of engineered reporter viruses containing site-directed polymorphic changes in Gag that reduce susceptibility to bevirimat (including V362I, V370A/M/Δ, and T371A/Δ), leading incrementally to the identification of BMS-955176. BMS-955176 exhibits potent activity (50% effective concentration [EC_50_], 3.9 ± 3.4 nM [mean ± standard deviation]) toward a library (*n* = 87) of *gag/pr* recombinant viruses representing 96.5% of subtype B polymorphic Gag diversity near the CA/SP1 cleavage site. BMS-955176 exhibited a median EC_50_ of 21 nM toward a library of subtype B clinical isolates assayed in peripheral blood mononuclear cells (PBMCs). Potent activity was maintained against a panel of reverse transcriptase, protease, and integrase inhibitor-resistant viruses, with EC_50_s similar to those for the wild-type virus. A 5.4-fold reduction in EC_50_ occurred in the presence of 40% human serum plus 27 mg/ml of human serum albumin (HSA), which corresponded well to an *in vitro* measurement of 86% human serum binding. Time-of-addition and pseudotype reporter virus studies confirm a mechanism of action for the compound that occurs late in the virus replication cycle. BMS-955176 inhibits HIV-1 protease cleavage at the CA/SP1 junction within Gag in virus-like particles (VLPs) and in HIV-1-infected cells, and it binds reversibly and with high affinity to assembled Gag in purified HIV-1 VLPs. Finally, *in vitro* combination studies showed no antagonistic interactions with representative antiretrovirals (ARVs) of other mechanistic classes. In conclusion, BMS-955176 is a second-generation MI with potent *in vitro* anti-HIV-1 activity and a greatly improved preclinical profile compared to that of bevirimat.

## INTRODUCTION

Infection with HIV-1 continues to be a serious health threat throughout the world, with more than 1 million infected individuals in the United States, nearly 40 million worldwide, and an estimated 2.1 million individuals worldwide becoming newly infected in 2014 (1, 2). Although >35 approved therapies have proven to efficiently suppress virus replication and resistance (3–5), there are still significant unmet medical needs in HIV-1 treatment (6) due to multidrug resistance development, as well as from long-term toxicities and comorbidities observed in patients using current treatment options (7, 8). Given this landscape, new drugs with novel mechanisms of action (MOAs) that can be used as part of a preferred regimen should still have a strong role to play in combination antiretroviral therapy (cART) regimens if they can be used as part of a once-a-day preferred regimen, have high genetic barriers to the development of resistance in the context of fixed-dose combinations and regimens, have improved safety over current agents, and have minimal drug-drug interactions.

**FIG 1:**
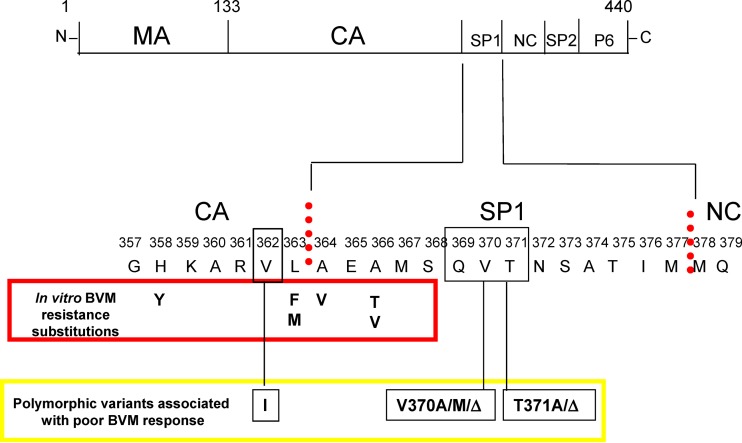
HIV-1 Gag polyprotein with the segments processed by HIV-1 protease, and amino acid differences that contribute to reduced BVM susceptibility. (Top) Gag with segments of the structural proteins cleaved by HIV-1 protease. (Bottom) HIV-1 Gag region surrounding SP1. Vertical dotted lines indicate protease cleavage sites; amino acids observed as resistance substitutions in *in vitro* selections reported for BVM are shown in the red box; A364V also reported as appearing as a resistance substitution in a BVM clinical study (58); black boxes show polymorphic amino acid positions within and near SP1 which reduce BVM susceptibility, with their variations identified in yellow box. Figure adapted from the work of Margot et al. (21).

The HIV-1 maturation process is essential for the production of infectious virions. It occurs through a series of HIV-1 protease-mediated cleavage reactions, with the last event occurring within the structural polyprotein Gag (Fig. 1), at a site between the capsid (CA) and spacer peptide 1 (SP1). This cleavage triggers a structural rearrangement, transforming the immature virus particle to a mature virion, characterized by an electron-dense conical core. Inhibition of this last cleavage step results in the release of immature, noninfectious virus particles (9, 10).

Bevirimat (BVM) was originally identified as a late-acting inhibitor of the viral life cycle (11). It binds to Gag (9) and blocks the last protease-mediated cleavage at the CA/SP1 boundary (10, 12–15). While BVM was safe and efficacious in humans, providing proof of concept for HIV-1 maturation as a valid antiviral target (16, 17), a phase IIa placebo-controlled study found that only 45% of patients (20/44) responded to treatment with a viral load reduction (VLR) of ≥0.5 log_10_. A retrospective analysis found reduced responses were associated with naturally occurring, so-called QVT (glutamine, valine, threonine) polymorphisms located near the CA/SP1 cleavage site (Fig. 1) in ∼50% of subtype B patient isolates (17–19) (Gag amino acids 369, 370, and 371). A single variation at amino acid 370 was shown to be a key determinant in mediating poor response of subtype C by BVM (20), while V362I was identified in subsequent studies (21) as an additional polymorphic variation that can affect BVM susceptibility. Consequently, due to the high prevalence of naturally occurring BVM resistance substitutions, together with its high protein binding and difficulties associated with formulation (22), BVM development was terminated. Other maturation inhibitors (MIs) structurally related to BVM (23–25) have been disclosed, with one reportedly active toward V370A, a key BVM-resistant polymorphism (26, 27), while another, more structurally diverse analog of betulinic acid entered phase 1 clinical trials (28). Another MI, PF-46396, is structurally distinct from BVM (29, 30), with a resistance profile that overlaps but is nonidentical to that of BVM (31).

The failure of BVM in the clinic primarily resulted from inadequate coverage of polymorphic variants in the general HIV-1 population. In addition, BVM exhibited high human serum binding, necessitating that high clinical exposures be reached. Armed with this knowledge, a strict set of screening guidelines were established in order to identify a second-generation MI. This incorporated screening for activity against key polymorphisms along with reduced human serum binding early in the discovery process. This allowed us to identify BMS-955176, a second-generation MI with broad-spectrum antiviral activity, low serum-adjusted potency, and the potential for once-a-day dosing (32–35). Here we report the antiviral and biochemical profiling of BMS-955176.

## MATERIALS AND METHODS

### Compounds.

BMS-378806, BMS-1, BMS-2, BMS-3, BMS-955176, bevirimat (BVM), raltegravir, BMS-817889 (a hepatitis C virus [HCV] NS5B inhibitor), atazanavir (ATV), lamivudine (3TC), abacavir (ABC), emtricitabine (FTC), nelfinavir (NFV), and efavirenz (EFV) were prepared at Bristol-Myers Squibb (BMS). Ritonavir (RTV), nevirapine (NVP), zidovudine (AZT), darunavir (DRV), tenofovir, and rilpivirine (RPV) were purchased and purified from commercial sources.

### Cells.

MT-2 cells, 293T cells, and HeLa-CD4 cells were obtained from the NIH AIDS Research and Reference Reagent Program. HeLa C14 cells expressing CD4, CXCR4, and CCR5 proteins and coexpressing firefly luciferase under the control of the tetracycline responsive element (TRE) were constructed at Bristol-Myers Squibb (36). B6, obtained from the DuPont Pharmaceutical Company, is an MT-4 cell line stably transfected with an HIV-1 long terminal repeat (LTR)-driven firefly luciferase reporter. Cell lines were subcultured twice a week in either RPMI 1640 (MT-2 and B6 cells) or Dulbecco modified Eagle medium (DMEM) (293T and HeLa cells) supplemented with 10% heat-inactivated fetal bovine serum (FBS), 100 U/ml of penicillin G, and 100 μg/ml of streptomycin. The DMEM was additionally supplemented with 10 mM HEPES buffer (pH 7.55), 2 mM l-glutamine, and 0.25 μg/ml of amphotericin B. Peripheral blood mononuclear cells (PBMCs) were isolated as described previously (37).

### Viruses.

The proviral plasmid pNLRepRluc was constructed at Bristol-Myers Squibb starting from a proviral NL_4-3_ clone (B subtype) obtained from the NIH AIDS Research and Reference Reagent Program. It contains the Renilla luciferase marker in place of the viral *nef* gene (38). P373S was subsequently introduced into this proviral clone (NLRepRlucP373S = wild type [WT]), since P373S is the most common variation at 373 within SP1 in subtype B. Other recombinant viruses were generated by site-directed mutagenesis of plasmid pNLRepRlucP373S to introduce amino acid substitutions representing relevant polymorphic variations in Gag. A panel of recombinant viruses was created by cloning *gag/protease* gene pools (*gag/pr*) from subtype B, C, and AE patient isolates (obtained from the NIH AIDS Research and Reference Reagent Program or from BMS-sponsored clinical trials [39, 40]) into pNLRepluc. Successful cloning was checked through sequencing of both the PCR product and the plasmid DNA. Recombinant viral DNAs were used to generate virus stocks by harvesting 2 to 3 days posttransfection in 293T cells (Lipofectamine PLUS kit; Invitrogen); titers were determined by luciferase activity in MT-2 cells, and virus stocks were stored at −80°C before use.

Laboratory-adapted HIV-1 strains RF, SF2, IIIB, HXB2, NL_4-3_, LAI, MN, Bal, JRFL, LAI M184V (3TC resistant), 7324-1 (AZT resistant), and IIIB-K103N/Y181C (efavirenz resistant) and laboratory-adapted HIV-2 strains ROD, CBL-20, and CBL-23 were obtained from the NIH AIDS Research and Reference Reagent Program. The raltegravir-resistant virus NLRepRluc-G140S/Q148H was constructed at Bristol-Myers Squibb. The *gag/pr* genes from a protease inhibitor (PI)-resistant virus (containing 46I, 82F, 84V, and 90M) were obtained from Nijhuis (UMC Utrecht) and transferred into the NLRepRluc backbone. The ritonavir- and atazanavir-resistant virus RF-RTV was as described previously (41). Titers of virus stocks were determined in their respective host cells (MT-2 for CXCR4-utilizing strains and PM1 for CCR5-utilizing strains), using as endpoints a virus infectivity assay (42) with either cytopathic effects (CPE) for all CXCR4-utilizing strains, a luciferase assay (dual-luciferase reporter assay system; Promega, Milwaukee, WI) for the reporter viruses, or a p24 antigen enzyme-linked immunosorbent assay (ELISA) (p24 ELISA kit; PerkinElmer Life Sciences, Waltham, MA) for the CCR5-utizing strains. The 50% tissue culture infective dose (TCID_50_) per milliliter was calculated by the Spearman-Karber method (43).

Clinical isolates (NIH AIDS Research and Reference Reagent Program) were amplified in freshly isolated and stimulated PMBCs. Titers of virus stocks were determined in PBMCs using a virus infectivity assay with a p24 antigen endpoint (p24 ELISA kit; PerkinElmer Life Sciences). The TCID_50_ per milliliter was calculated by the Spearman-Karber method (43).

### Cytotoxicity assays.

Concentrations of BMS-955176 required to reduce cell viability by 50% (CC_50_s) were determined in multiple cell lines using a respiration assay (MTS; Promega) according to the manufacturer's protocol (44) as described in the supplemental material.

### Multiple-cycle drug susceptibility assays.

Pellets of MT-2 cells were infected with NLRepRlucP373S Gag variants containing clinical isolate *gag/pr* genes or site-directed mutations, or cells were infected with a panel of HIV-1 CXCR4-tropic and HIV-2 laboratory strains. Pellets of PM1 cells were infected with either Bal or JRFL virus. Initial inocula of the reporter strains were normalized using equivalent endpoint luciferase activity signals, whereas the reporter-free strains were tested at an initial multiplicity of infection (MOI) of 0.005. Cell-virus mixtures were resuspended in medium, incubated for 1 h at 37°C and 5% CO_2_, and added to compound-containing 96-well plates at a final density of 10,000 cells per well. Test compounds were 3-fold serially diluted in 100% dimethyl sulfoxide (DMSO) and assayed at a final DMSO concentration of 1%. All assays included 1% DMSO as a “no drug, no inhibition” control and were used in data analysis. After 4 to 5 days of incubation at 37°C and 5% CO_2_, virus yields were determined by either Renilla luciferase activity (dual-luciferase reporter assay system; Promega) for the reporter virus, p24 yield (p24 ELISA kit, PerkinElmer Life Sciences) for the CCR5-tropic virus, or reverse transcriptase (RT) activity for the CXCR4-tropic virus. RT activity was measured in a scintillation proximity assay (SPA) as described previously (45).

Serum effects in multiple-cycle drug susceptibility assays were determined by supplementing the 10% FBS medium with 40% human serum (HS) containing 27 mg/ml of additional human serum albumin (HSA), 45 mg/ml of HSA, 40% HS plus 1 mg/ml of α-acid glycoprotein (AGP), or 45 mg/ml of HSA plus 1 mg/ml of added AGP. The serum effect was calculated as the fold change in EC_50_ versus the no-serum EC_50_.

The 50% inhibitory concentrations (EC_50_s) for cell culture assays were calculated by using the exponential form of the median effect equation where percent inhibition = 1/[1 + (EC_50_/drug concentration)*m*], where *m* is a parameter that reflects the slope of the concentration-response curve. The background was taken as the residual signal observed upon inhibition at the highest concentration of a control protease inhibitor, NFV (3 μM). Fold change EC_50_ (FC-EC_50_) values were calculated as the EC_50_ change versus WT EC_50_.

### Single-cycle drug susceptibility assays.

In an adaptation of a published assay (46), in the first step, 1.5 μg of full-length recombinant pNLRepRuc DNA variants and 1.5 μg of plasmid SV-A-MuLV-env (murine leukemia virus [MuLV] envelope gene under the control of the simian virus 40 [SV40] promoter, obtained from the NIH AIDS Research and Reference Reagent Program; catalog number 1065) were cotransfected (calcium phosphate; Life Technologies) into 293T cells (60 to 70% confluence; 6-well plate). After an overnight incubation at 37°C and 5% CO_2_, transfected cells were washed, trypsin treated, and then coseeded with untransfected 293T cells onto compound-containing 384-well plates at the cell number ratio of 1:5 to a final cell density of 9,500 cells/well. After 72 h of incubation (37°C and 5% CO_2_), cell-associated Renilla luciferase activity was measured upon the addition of EnduRen live cell substrate (Promega). EC_50_s were calculated as for the multiple-cycle assay.

### Drug susceptibility assays using clinical isolates.

Pellets of PBMCs, stimulated with interleukin 2 (IL-2) and phytohemagglutinin (PHA-P) for 2 days, were infected with clinical isolates at an MOI of 0.005 and incubated in 0.5 ml of medium at 37°C and 5% CO_2_ for 3 h, prior to resuspension in medium and addition to 96-well plates containing 3-fold serially diluted compounds. The final cell density was 1 × 10^6^/ml. Virus yields were monitored from day 5 postinfection (p24 antigen ELISA; PerkinElmer Life Sciences, Waltham, MA), and assays were terminated when 1,000-fold-diluted supernatants of the control infection (no drug) yielded a level of p24 within a dynamic range (0.6 ≤ *A*_490_ ≤ 2.0).

### HIV-1 entry assay.

Confirmation that BMS-955176 functions only at a late stage in the infectious cycle was obtained by a two-stage single-cycle experiment targeting early and late stages in the replication cycle (luciferase readout using MuLV-pseudotyped virus by a variation of a previously reported method [46]). Inhibition of early stages includes events up to integration, while inhibition of late stages includes viral assembly and maturation. In the first step, 10 μg of envelope deletion plasmid pNLRepRucP373SΔ*env* (RepRlucP373S with deletion of 500 bp internal to *env*) and 10 μg of plasmid HIV-1 pLAI-*env* were cotransfected (calcium phosphate; Life Technologies) into 293T cells. After overnight incubation at 37°C and 5% CO_2_, the transfected cells were washed, trypsin treated, and resuspended in fresh medium at a density of 1.0 × 10^5^/ml. Cells (100 μl/well) were then distributed into 96-well plates that contained 3-fold serially (DMSO) diluted compound in 100 μl of medium (final DMSO concentration, 1%). After ∼30 h of incubation at 37°C/5% CO_2_, 100 μl of supernatant (containing the newly produced virus but no cells) was transferred to a new 96-well plate seeded with MT-2 cells (3.5 × 10^4^/well). After a 2-day incubation, MT-2 cell-associated Renilla luciferase activity was measured upon the addition of EnduRen live cell substrate (Promega), using an EnVision multilabel reader (PerkinElmer; product number 2104-0010).

### Cell-based Gag p25 cleavage assays.

BMS-955176 was evaluated for its ability to inhibit the final step of HIV-1 protease cleavage of the Gag polyprotein in the context of HIV-1-infected cells as follows. 293T cells were treated with BMS-955176 (200 nM or 0 nM) and then transfected (TransIT-LT1 transfection reagent MIR 2300; Mirus, Bio LLC) with pNLRepRlucP373S or pNLRepRlucP373S/A364V DNA. Two days posttransfection, supernatants were concentrated over a 20% sucrose cushion (14,000 × *g* for 30 min) and analyzed for HIV-1 Gag cleavage products by Western analysis using a primary anti-p24 monoclonal antibody (PerkinElmer; NEA9306001) and a secondary goat anti-mouse antibody (RPN2132; Bio-Rad, Hercules, CA) and developed with the ECL Plus detection kit (Amersham, Marlborough, MA). Intensities of p25 (25 kDa) and p24 (24 kDa) cleavage products were quantified using a STORM 860 phosphorimager (Molecular Dynamics, Sunnyvale, CA).

### Preparation of HIV-1 VLPs.

Noninfectious HIV-1 virus-like particles (VLPs) (47, 48) containing only the Gag structural protein (49, 50) were expressed from a synthetic gene under the control of the cytomegalovirus (CMV) promoter (plasmid 1_pcDNAGagOpt, constructed at Bristol-Myers Squibb). GagOpt encodes the full-length HIV-1 LAI Gag protein, with codons optimized for expression in mammalian cells, encompassing the coding sequence starting from the N terminus of matrix (MA; amino acid position 1) and extending to the stop codon of p6. A364V 1_pcDNAGagOpt contains the A364V mutation resistant to BVM (51, 52). HIV-1 Gag VLPs were prepared by transfection of 1_pcDNAGagOpt derivatives (18 μg) in 293T cells. Two days posttransfection, culture supernatants were filtered (0.45-μm filter; Millipore, Darmstadt, Germany) and VLPs pelleted for 2 h (20% sucrose cushion at 25,000 rpm). Pellets were resuspended in phosphate-buffered saline (PBS) at a total protein concentration of about 1,000 μg/ml (stored at −80°C).

### HIV-1 VLP protease cleavage assay.

Purified WT VLPs or A364V VLPs (300 ng) were incubated at 0°C for 10 min in 30 μl of VLP buffer (50 mM morpholineethanesulfonic acid [MES; pH 6.0], 100 mM NaCl, 2 mM EDTA) supplemented with 0.06% Triton X-100 to remove the lipid bilayer, and the delipidated VLPs were then diluted 10-fold in cold VLP buffer. Ten nanograms of delipidated VLPs was incubated at 24°C for 2 h with serial dilutions of BMS-955176 or BVM and then digested with 2.9 μM HIV-1 protease at 30°C for 60 min. The protease used contained substitutions that limit autoproteolysis (Q7K, L10I, I13V, L33I, S37N, R41K, L63I, C67A, and C95A), which allows for complete digestion of the VLPs (53). Samples were analyzed as described for those produced by cell-based Gag p25 cleavage assays in cells.

### [^3^H]BMS-955176-Gag binding.

Specific binding of [^3^H]BMS-955176 to Gag was demonstrated using a scintillation proximity assay (SPA), which is a radiolabeled binding assay. A total of 0.5 μg of WT VLPs (PBS solution) was mixed with 100 μg of SPA beads (PBS suspension; PVT WGA SPA beads; PerkinElmer) in a total volume of 40 μl per well (96-well white low-binding plate [Corning, Corning, NY]). After a 1-h incubation at room temperature, the volume was increased to 180 μl/well by the addition of binding buffer (100 mM Tris [pH 6.5], 2 mM EDTA, 0.03% Tween 20, 5 mM MgCl_2_). A *K_d_* (dissociation constant) determination was made by adding 20 μl of a serial dilution of [^3^H]BMS-955176 (0.2 to 600 nM) to the VLP mixture. The data were fit to an equation for saturation binding (GraphPad v 5.1). For determination of a *K_i_* value, 11 nM [^3^H]BMS-955176 was added to the VLP-bead mixtures, to which was added a serial dilution of unlabeled BMS-955176 or BVM. After 4 h of equilibration at room temperature, bound [^3^H]BMS-955176 was measured using a Top Count plate reader (PerkinElmer). The data were fit to an equation for homologous competition (GraphPad v 5.1).

See the supplemental material for experimental details concerning the following: cytotoxicity assay, off-target activity assays, two-drug combination assays, HIV-1 cell fusion assay, and ultracentrifugation serum binding assay.

## RESULTS

### BMS-955176 exhibits potent activity toward key BVM-resistant Gag polymorphisms in cell culture.

**FIG 2:**
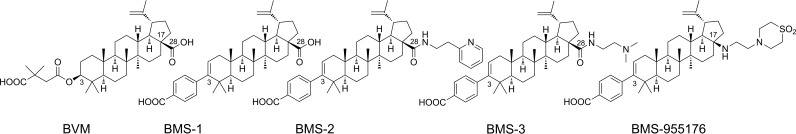
Structures of maturation inhibitors.

A structure-activity relationship (SAR) strategy was driven using a panel of BVM-resistant Gag polymorphic site-directed mutant (SDM) viruses, including V362I, V370A, and V370Δ mutants, with concomitant evaluation of serum effects toward WT virus. Testing was performed using multiple-cycle drug susceptibility assays with Renilla luciferase activity as the assay endpoint. Promising candidates were further profiled against a larger cohort of SDMs, results for which are shown in Table 1 (additional details are in the supplemental material). The series illustrates successive improvements in potency against the various polymorphisms by molecules BMS-1, BMS-2, and BMS-3, compared to BVM, leading ultimately to the identification of BMS-955176 (Fig. 2) (54–56).

**TABLE 1:** SAR progression toward clinical candidate BMS-955176: coverage of BVM-resistant Gag polymorphisms^*a*^

HIV-1 NL_4-3_	Subtype B, LANL, %^*b*^	EC_50_ (SD), nM					FC-EC_50_	
		BVM	BMS-1	BMS-2	BMS-3	BMS-955176	BVM	BMS-955176
WT	51	**10** (11)	16 (13)	15 (13)	**2.3** (1.3)	**1.9** (1.8)		
WT in HS^*c*^		1,291 (1,011)	105 (40)	153 (269)	43 (29)	10.2 (6.0)	130	5.4
V362I variant	9.8	74 (59)	213 (170)	76 (34)	18 (26)	**4.5** (2.2)	7.4	**2.4**
Q369H variant	1.2	**7.0** (2.0)	ND	ND	ND	**1.9** (0.9)	**0.64**	**1.0**
V370A variant	12.4	552 (633)	233 (305)	19 (13)	**7.8** (8.8)	**2.7** (1.5)	54	**1.4**
V370M variant	4.4	1,810 (1,900)	>4,000	61 (31)	**10** (8.0)	**2.8** (0.3)	177	**1.5**
ΔV370 variant^*d*^	0.9	>10,000	>6,000	415 (313)	31 (19)	**13** (11)	>1,000	**6.8**
V370A/ΔT371 variant^*d*^	1.4	1,114 (1,197)	>10,000	188 (105)	ND	**6.6** (3.9)	109	**3.5**
T371A variant	0.4	40 (48)	28 (22)	19 (14)	**3.4** (2.7)	**2.0** (0.1**)**	**3.9**	**1.0**
ΔT371 variant	4.0	77 (97)	292 (234)	38 (27)	**3.0** (1.6)	**7.3** (3.9)	7.5	**3.8**
A364V variant	0	>10,000	>10,000	740 (670)	>10,000	1,480 (740)	>1,000	759

a Abbreviations: FC-EC_50_, EC_50_ of test assay/EC_50_ of WT virus (NLRepRlucP373S). Values are means (SD) from experiments performed at least three times; additional details are in Table S1 in the supplemental material. BVM, bevirimat; ND, not determined; WT, wild type. Cytotoxicity of BMS-955176 was examined in five cell lines (details are provided in the supplemental material), and the results were suggestive of moderate cytotoxicity, while the therapeutic index was MT-2 cells was relatively high (4,842), indicating that the effects of BMS-955176 are virus specific. In addition, in initial clinical studies, BMS-955176 has been safe and well tolerated (32, 34, 70). Boldface indicates EC_50_ values < 10 nM.

b Percent key single amino acids is the percentage not in consensus with the WT (HIV-1 HXB2) at one of four Gag positions (V362, Q369, V370, and T371), with concurrent HXB2 amino acids at the remaining three positions (filtered alignments of the Los Alamos National Laboratory [LANL] database, 2012; *n* = 1,642).

c Compounds were evaluated for serum effects in 10% FBS plus 40% human serum except for BMS-955176 (10% FBS plus 40% human serum plus 27 mg/ml of human serum albumin).

d Surrogate for subtype C.

As seen in Table 1, the majority (6/8) of the polymorphic viruses display lower susceptibility to BVM than to the other betulin derivatives. Replacement of the C3-O-3′,3′-dimethylsuccinyl group in BVM with a benzoic acid moiety (BMS-1; Fig. 2) did not significantly affect potency against the WT (from 10 to 16 nM, respectively) but resulted in a substantial reduction of the effect of human serum on potency, from a 130-fold increase for BVM to only a 7-fold increase for BMS-1. Therefore, the benzoic acid moiety was preserved in successive compounds. Additional modifications to the right-hand side of the molecule at C28 provided BMS-2 (C28 pyridyl amide) and BMS-3 (C28 dimethylaminoethyl amide). These compounds also exhibited lower serum shifts while exhibiting improvements in potency against the V370 (V370A/M, ΔV370, and V370A/ΔT371) polymorphisms. BMS-3 has a relatively flat and potent profile, with an EC_50_ of 31 nM for the ΔV370 polymorph and a 19-fold serum shift. Further modifications at C28 provided BMS-955176 (N17 aminoethyl thiomorpholinosulfone), which is ∼5-fold more active toward WT virus than BVM (EC_50_ of 1.9 nM versus 10 nM) and continues to have a reduced serum shift (5.4-fold). BMS-955176 also exhibits potent antiviral activity toward key Gag polymorphisms, with EC_50_s of <5 nM against V362I, Q369H, V370A/M, and T371A/Δ viruses. Against the highly BVM-insensitive V370A/ΔT371 and ΔV370 polymorphic viruses (both characteristic of subtype C) (20), BMS-955176 exhibits low EC_50_s, 6.6 and 13 nM, respectively. The relevance of this improvement in potency is highlighted by the fact that several of these key polymorphisms are relatively common, with HIV-1 subtype B prevalences of single polymorphisms of 12.4% (V370A), 9.8% (V362I), and 4.4% (V370M), according to the 2012 version of the filtered web alignments of the Los Alamos National Laboratory (LANL) HIV-1 Sequence Database (57). The virus variant with the lowest susceptibility in this panel is A364V, which is not found in circulating viruses but was selected by BVM, both *in vitro* (58, 59) and *in vivo* (60). The BMS MIs in this study all exhibited reduced activity toward the A364V virus (Table 1).

### BMS-955176 exhibits moderate HS binding and HS effects in antiviral assays.

The effect of human serum (HS) components on BMS-955176 antiviral potency was assessed in cultures of MT-2 cells infected with the NLRepRlucP373S WT virus (see Table S2 in the supplemental material), using luciferase activity as the assay endpoint. To mimic the effect of 100% serum without compromising cell viability, 10% FBS medium was supplemented with both 40% HS and 27 mg/ml of human serum albumin (HSA). This brought the overall concentration of HSA in the culture medium to the physiological concentration of 45 mg/ml. Under these conditions (10% FBS plus 40% HS plus 27 mg/ml of HSA), BMS-955176 exhibited a 5.4-reduction of antiviral activity compared to that with 10% FBS (EC_50_ of 10.2 nM versus 1.9 nM, respectively). HSA seemed to partially contribute to the serum effect, since it produced a 2.5-fold reduction in potency when added alone at its physiological concentration of 45 mg/ml. A similar 2.6-fold shift was observed with 40% HS alone. The addition of 40% serum in the absence of MI decreased viral infectivity somewhat, but control experiments established that this did not interfere with determination of serum effects. Conversely, the effect of physiological levels (1 mg/ml) of α-acid glycoprotein (AGP) is negligible (EC_50_ of 1.0 nM versus 1.9 nM for 10% FBS). In agreement with this observation, the addition of 1 mg/ml of AGP to 45 mg/ml of HSA in the 10% FBS medium did not substantially change potency (EC_50_ of 6.2 nM versus 4.7 nM). These results suggest that BMS-955176 binds weakly to HSA and perhaps another component(s) in serum but that AGP is not involved. A 5.4-fold serum shift implies human serum binding of 82%, which corresponds well with a value of 86% measured by an ultracentrifugation method (see the supplemental material) (61).

### BMS-955176 exhibits potent activity toward HIV-1 laboratory strains.

BMS-955176 was subsequently examined against a panel of laboratory strains, including both CXCR4 strains (evaluated in MT-2 cells: RF, SF-2, IIIB, HXB2, NL_4-3_, LAI, and MN) and CCR5 strains (evaluated in PM1 cells: Bal and JRFL). The results in Table 2 confirm that BMS-955176 maintains its potency regardless of coreceptor usage. The EC_50_s ranged from 0.7 nM (Bal) to 11 nM (SF-2 and HXB2). MJ4, a subtype C molecular clone, was evaluated by RT activity in PBMCs and provided an EC_50_ of 3 nM.

**TABLE 2:** Activity of BMS-955176 against HIV-1 and HIV-2 laboratory strains

Virus	Coreceptor tropism	Strain	Host cells	EC_50_ (nM)^*a*^
HIV-1	CXCR4	RF	MT-2	7.7 ± 1.4
		SF-2	MT-2	11 ± 1.5
		IIIB	MT-2	2.8 ± 0.3
		HXB2	MT-2	11 ± 2.0
		NL_4-3_	MT-2	4.7 ± 2.3
		LAI	MT-2	2.6 ± 0.3
		MN	MT-2	0.9 ± 0.6
	CCR5	Bal	PM1	0.7 ± 0.2
		JRFL	PM1	2.5 ± 0.8
HIV-2	CXCR4	ROD	MT-2	15 ± 1.2
		CBL-20	MT-2	>1,200
		CBL-23	MT-2	>1,200

a Values are means ± SD of experiments performed a minimum of 3 times.

BMS-955176 was also tested against three HIV-2 strains (ROD, CBL-20, and CBL-23) in MT-2 cells (Table 2). While HIV-2 ROD was inhibited with an EC_50_ of 15 ± 1.2 nM, the other two strains were completely resistant to BMS-955176. There are a total of 12 Gag amino acid changes between ROD and the two CBL strains. Interestingly, three changes, at positions 370, 371, and 372, are located near the purported site of action of BMS-955176. At this time, it is not known which differences are responsible for the susceptibility of ROD to BMS-955176 (see Table S3 in the supplemental material).

### BMS-955176 is a late inhibitor of HIV-1 replication.

**FIG 3:**
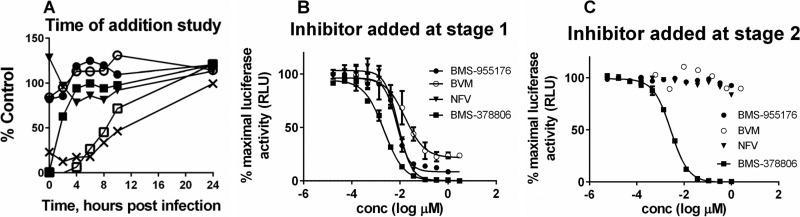
Effect of BMS-955176 on virus replication within a single-cycle assay. (A) Time of addition study. Changes in the relative level of viral infection in cell culture 24 h postinfection were measured by firefly luciferase activity as a function of the time of addition of the following inhibitors: BMS-378806 (AI; closed squares), efavirenz (NNRTI; open squares), raltegravir (INSTI; crosses), nelfinavir (PI; closed triangles), BVM (MI; open circles), and BMS-955176 (MI, closed circle). The final concentrations of each inhibitor were as follows: BMS-378806, 0.36 μM; raltegravir, 0.73 μM; efavirenz, 0.5 μM; nelfinavir, 1 μM; BVM, 1 μM; and BMS-955176, 1 μM. (B and C) Antiviral profiles of selected ARVs of differing mechanisms in a single-cycle assay, with test compound added at stage 1 (B) or stage 2 (C) as described in the text. Test compounds included BMS-955176 (filled circles), nelfinavir (NFV; triangles), BMS-378806 (squares), and BVM (open circles). Panel A is a representative example of one of two studies giving similar results. Panels B and C show mean values, with standard deviations, for a representative experiment performed in triplicate. RLU, relative light units.

In order to determine the point within the virus life cycle at which BMS-955176 exerts its activity, a time-of-addition (TOA) study was performed during a single cycle of virus replication (Fig. 3A). Briefly, NL_4-3_-infected B6 cells were treated with test inhibitors representing various mechanistic drug classes at different time points postinfection. Drug concentrations (>100-fold EC_50_s) were chosen such that complete inhibition should be observed if added at a time prior to its drug action. The level of virus replication was monitored by activity of cell-encoded firefly luciferase, expressed from a Tat-driven LTR promoter upon virus integration. Figure 3A shows the percentage of luciferase signal remaining 24 h postinfection upon treatment at different time points with various drugs. In this study, the attachment inhibitor (AI) BMS-378806 was fully inhibitory (0% of control) only when added at the time of infection, and it lost its effectiveness within the first 4 h postinfection, which agrees with its virus entry mode of inhibition (62). In contrast, efavirenz and raltegravir began to lose their effectiveness beginning at 6 and 8 h postinfection, respectively. This is in accord with the mechanisms of action of nonnucleoside reverse transcriptase inhibitors (NNRTIs) and integrase strand transfer inhibitors (INSTIs), which act before virus integration (63). As expected, two late inhibitors, the PI nelfinavir (NFV) and the MI BVM, both inhibiting postintegration, had no effect when added at any time point. In a similar manner, BMS-955176 also did not inhibit luciferase expression when added any time during infection. This result indicates that BMS-955176 acts as a late inhibitor, subsequent to proviral integration and transcription.

In order to confirm that BMS-955176 is a late inhibitor of HIV-1 infection, a variation of a two-stage single-cycle pseudotype assay was performed (46). In stage 1, HIV-LAI envelope-pseudotyped RepRlucP373SΔ*env* virus was prepared by cotransfection of pLAI*env* (64) and RepRlucP373SΔ*env* plasmids into HEK293T cells. Subsequently, the supernatant was transferred to a plate which contains MT-2 cells for infection (stage 2). Expression of luciferase in the infected cell (stage 2) indicates that infectious virus had been produced in the virus production stage (stage 1). Inhibitors of virion maturation and assembly should inhibit during the virus production stage, while an attachment inhibitor should inhibit only during the infection stage (stage 2). Therefore, two experiments were performed with the addition of the drugs (BMS-955176, BVM, nelfinavir, and BMS-378806) at different stages. When the protease inhibitor NFV was added in the virus production stage (stage 1), luciferase activity was inhibited (Fig. 3B). However, when NFV was added only at stage 2, the infection stage, luciferase production was not inhibited (Fig. 3C). An HIV-1 attachment inhibitor (AI; BMS-378806) was inhibitory when added at either stage 1 or 2 (Fig. 3B and C). AI might be expected to inhibit only during the infection stage (stage 2); however, AI inhibits in both cases due to carryover of supernatant from stage 1 to stage 2 (50% of the volume). BMS-955176 and control MI BVM behaved similarly to nelfinavir, inhibiting luciferase production only when added in the first stage of the assay (cf. Fig. 3B and not C), consistent with a late mechanism of action. In this single-cycle format, BVM (Fig. 3B) reproducibly does not attain complete inhibition relative to NFV (under further study).

HIV-1 entry inhibition, previously shown for certain betulinic acid derivatives (65), was further excluded for BMS-955176 in a cell-cell fusion assay (64), facilitated by HIV-1 gp160, which is expressed on the surface of donor cells, resulting in activation of luciferase expression in target cells. Such fusion was efficiently inhibited by BMS-378806, with an EC_50_ of 0.9 nM, whereas both NFV and BMS-955176 were inactive, with EC_50_s of >2 μM (data not shown). Overall, the results confirm that BMS-955176 does not exhibit any inhibition at an early stage of infection and functions purely as a late inhibitor.

### BMS-955176 inhibits Gag cleavage at CA/SP1.

**FIG 4:**
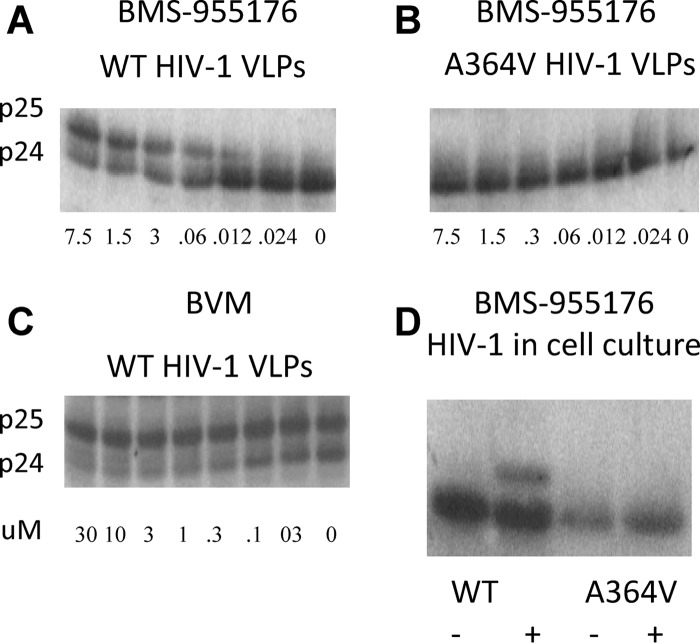
Inhibition of cleavage of HIV-1 p25 to p24. (A to C) Inhibition of HIV-1 p25 cleavage of HIV-1 VLPs *ex cellulo*. Values are micromolar. (A) BMS-955176, wt VLPs; (B) BMS-955176, A364V VLPs; (C) BVM, WT VLPs; (D) HIV-1 in cells. The results are from one of two representative experiments. −, no BMS-955176; +, 200 nM BMS-955176.

BMS-955176 was evaluated *in vitro* for its ability to inhibit the final step of Gag cleavage, which results in the conversion of capsid (CA) precursor p25 to capsid p24 and spacer peptide 1 (66). Assembled HIV-1 virus-like particles (VLPs) were produced in 293T cells from a codon-optimized expression vector (1_pcDNAGagOpt) and were used as the substrate for cleavage by recombinant HIV-1 protease *in vitro*. As shown in the Western blots in Fig. 4A and C, BMS-955176 and BVM inhibited the conversion of HIV-1 WT VLP p25 to p24 in a dose-dependent manner. Conversely, BMS-955176 did not inhibit the cleavage of HIV-1 VLPs containing substitution A364V (Fig. 4B). Amino acid A364 is adjacent to the p25/p24 cleavage site (L363/A364), and the change of A to V imparts a loss of susceptibility to BMS-955176 (Table 1), as has been reported for *in vitro* selections for BVM resistance (51) and also reported in the BVM POC study (60). In another experiment, the ability of BMS-955176 to inhibit p25 cleavage within infected cells was probed. 293T cells were treated with 200 nM BMS-955176 and transfected with pNLRepRlucP373S or pNLRepRlucA364V/P373S. Two days posttransfection, cells were harvested and the cleavage of p25 was probed in a Western blot. As shown in Fig. 4D, BMS-955176 partially inhibited p25-to-p24 conversion in pRepRlucP373S (WT)-transfected 293T cells but did not inhibit the p25 cleavage of the HIV-1 strain containing the Gag A364V substitution. As shown in Fig. S6 in the supplemental material, there was no evidence that any other cleavage sites in Gag were inhibited by BMS-955176. In addition, a novel liquid chromatography-mass spectrometry (LC/MS) format was used to demonstrate specific inhibition of CA/SP1 by BMS-955176 and BVM (33), further details of which will be reported elsewhere. Altogether, the data for VLPs and HIV-1 infected cells indicate that BMS-955176 specifically inhibits the cleavage of capsid precursor p25 (CA/SP1) to capsid p24.

### BMS-955176 binds with high affinity and specificity to HIV-1 Gag.

**FIG 5:**
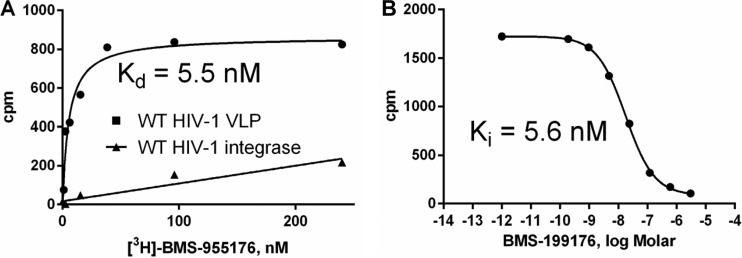
Binding of [^3^H]BMS-955176 to HIV-1 Gag VLPs (A) Binding of [3H]BMS-955176 to WT HIV-1 VLP (circles, nonspecific binding to integrase subtracted) and HIV-1 integrase (control). (B) Inhibition of [^3^H]BMS-955176 binding to WT VLPs by nonradiolabeled BMS-955176. Results are representative of 5 experiments to determine *K_d_* values with range of 2.3 to 6 nM.

Specific binding of BMS-955176 to HIV-1 Gag was demonstrated with a radiolabeled compound. The *K_d_* for the binding of serially diluted [^3^H]BMS-955176 to LAI VLPs *in vitro* was 5.5 nM (Fig. 5A) (33). The use of HIV-1 integrase in the assay in place of Gag VLPs resulted in only nonspecific nonsaturable binding (Fig. 5A). Specific binding of [^3^H]BMS-955176 was reduced in a dose-dependent manner by nonradiolabeled BMS-955176, with a *K_i_* of 5.6 nM (Fig. 5B). A similar result was obtained by displacement by other MIs, such as BVM (data not shown). On the other hand, a 10 μM concentration of a structurally and mechanistically unrelated HIV-1 inhibitor (raltegravir) did not compete for the binding of [^3^H]BMS-955176 (data not shown). These data indicate that BMS-955176 binding to HIV-1 Gag is specific, saturable, reversible, and competitive with BVM binding.

Thus, the inhibition of Gag cleavage and compound binding results support the proposed BMS-955176 mechanism of action. In this model, similarly to BVM, the compound binds to the assembled form of Gag and prevents the last step of Gag processing by the viral protease, resulting in inhibition of virus replication. Additionally, the target specificity of BMS-955176 is supported by negative results in biochemical counterscreening assays: an *in vitro* HIV-1 reverse transcriptase assay (50% inhibitory concentration [IC_50_] > 50 μM), influenza virus polymerase assay (IC_50_ >100 μM), an HIV-1 integrase inhibitor binding assay (IC_50_ >15 μM), and HIV-1 protease assay (IC_50_ > 10 μM). Overall, the results of these mechanistic studies, including specific Gag binding, inhibition of p25 cleavage, late mode of action in time-of-addition studies, and no off-target activities in the counterscreen, were consistent with the assignment of BMS-955176 as an HIV-1 maturation inhibitor.

### Predecessor MIs and BMS-955176 display improving potency against broad panels of subtype B and C clinical isolates in a multiple-cycle Gag/Pr phenotyping assay.

**FIG 6:**
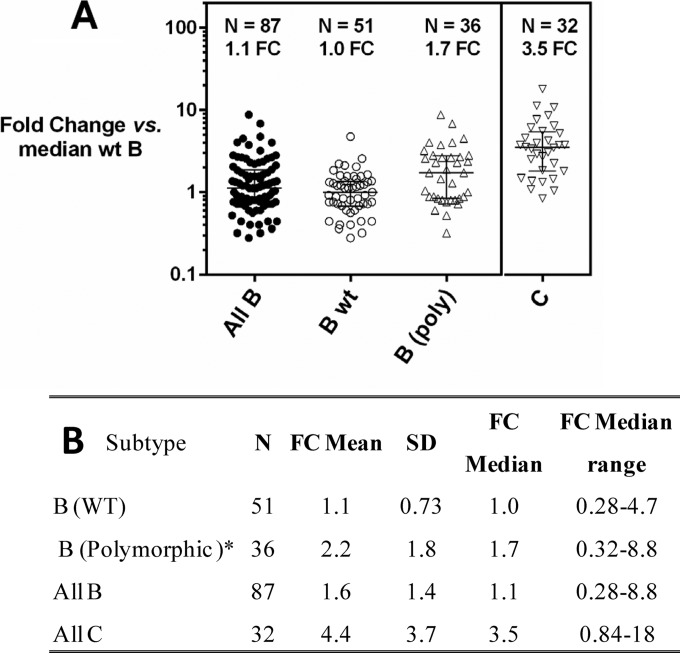
Evaluation of Gag/Pr libraries derived from subtype B and C clinical isolates for BMS-955176 sensitivity in a multiple-cycle assay. (A) Multiple-cycle assay comparing responses of panels of all subtype B (closed circles) and C (open inverted triangles) *gag/pr* recombinant viruses for BMS-955176 sensitivity. Polymorphic subtype B (open triangles) is defined as a single change at Gag position 362, 369, 370, or 371 from WT subtype B (open circles). The median EC_50_ for WT subtype B is 2.5 nM; bars are interquartiles of medians. (B) Statistics for panel A; FC-EC_50_ = EC_50_ test assay/median value of EC_50_ for library of 51 WT (no changes to Gag 362, 369, 370, or 371) *gag/pr* recombinant viruses. *, 36/87 total subtype B viruses had key a single polymorphism in Gag at position 362, 369, 370, or 371. Each experimental point is the mean of triplicate determinations.

The anti-HIV-1 spectrum of predecessor MIs and BMS-955176 (Table 3) were further assessed with a library of *gag/pr* recombinants in a multiple-cycle assay. *Gag/pr* amplicons from a collection of 119 clinical isolates representative of Gag and Pr polymorphic diversity in subtype B and C viruses (87 subtype B and 32 subtype C) were subcloned into a replication-competent reference backbone virus (NLRepRluc). Sequence analysis of Gag amino acids 357 through 380 in the cohorts (region of determinants of BVM sensitivity) (17, 59, 67) revealed that all but 2 contained multiple substitutions in this region, compared to the subtype B HXB2 consensus sequence. For the subsequent analysis, WT virus is defined as any virus with the following 4 Gag residues: V362, Q369, V370, and T371. The prevalence of single polymorphisms observed in the subtype B cohort closely mirrored that observed in the 2012 LANL database (data not shown), which confirms its close representation of the natural variability observed in this Gag region. The MI-specific genotypes of the 87 subtype B viruses and their susceptibilities to BMS-955176 are shown in Table 3 (column 6), and the distribution of BMS-955176 FC-EC_50_ values is shown in Fig. 6A.

**TABLE 3:** SAR progression toward clinical candidate BMS-955176: coverage of Gag/Pr libraries derived from subtype B clinical isolates^*a*^

Key single^*a*^ polymorphism(s)^*b*^	*n*^*c*^	EC_50_, nM			BMS-955176	
		BVM^*c*^	BMS-2^*c*^	BMS-3^*c*^	*n*	EC_50_, nM^*d*^
None (WT)	21	14	14	3	51	3
V362I	3	36	29	6	5	5
Q369H	1	4	7	15	1	2
V370I/L/T	3	251	26	10	4	3
V370A	3	227	38	12	7	2
V370 M	5	903	76	67	9	7
T371A/Q/N/S/TT	4	113	49	7	6	4
V370A + ΔT371	3	>4,000	629	236	4	12

a WT, no change at Gag amino acid position V362, Q369, V370, or T371 relative to HXB2; SAR: structural-activity relationship. Values are means from experiments performed in triplicate; coefficients of variation for all experiments were ≤200%.

b Key single polymorphisms are amino acids not in consensus (HIV-1 HXB2) at one of four Gag positions (V362, Q369, V370, and T371), with concurrent HXB2 amino acids at remaining three positions.

c Sublibrary, *n* = 43/87 (column 2).

d EC_50_ values are means.

In addition, a subgroup of 43 viruses (Table 3, column 2) was also analyzed against BVM and earlier BMS MIs, BMS-2 and BMS-3. The results for the predecessor MIs with respect to their activities in the context of the more complex Gag substitution patterns of the sublibrary (Table 3) mirror the results observed for the panel of Gag site-directed mutants (Table 1), i.e., progressive improvements to potency toward the polymorphic viruses.

The 87 subtype B variants represent 96.5% of the diversity in this region in subtype B, as found in the LANL database (57). BMS-955176 retained WT activity toward viruses harboring changes to Q369 (369 = H), V370 (370 = A, I, L, or T), T371 (371 = A, Q, N, or S or an insertion of T), and a set of five V362I viruses. Compared to BVM, viruses with polymorphic substitutions at positions 362, 370, and 371 were substantially more sensitive to BMS-955176. The V362I-containing viruses in Table 3 (*n* = 5) displayed similar BMS-955176 sensitivities (within 2-fold; EC_50_, 5.3 [mean]; median EC_50_, 5.7 nM; range, 2.0 to 9.4 nM) to that of the overall library of subtype B viruses. The mean EC_50_ for this panel toward BMS-955176 was 3.9 ± 3.4 nM, with a median value of 2.8 nM and a range from 0.7 nM to 22 nM (data not shown). The absolute EC_50_s of the data could also be normalized against the WT control virus (NLRepRlucP373S), and fold changes in EC_50_ could be determined (FC-EC_50_s = EC_50_ of test assay/EC_50_ NLRepRlucP373S). This provides a more controlled comparison across the cohort. The mean FC-EC_50_ for subtype B viruses (*n* = 87) was 1.6 ± 1.4, with a median FC-EC_50_ of 1.1, with a range of 0.28 to 8.8 (Fig. 6B). The mean FC-EC_50_ for subtype C viruses (*n* = 32) was 4.4 ± 3.7, with a median value of 3.5 and a range from 0.84 to 18. The subtype C median FC-EC_50_ was ∼3.5-fold higher than that for subtype B (Fig. 6). Altogether, these data indicate that BMS-955176 is distinguished from first-generation MIs, with superior potency toward subtypes B and C.

### BMS-955176 displays activity toward a broad panel of clinical isolates in PBMCs.

**FIG 7:**
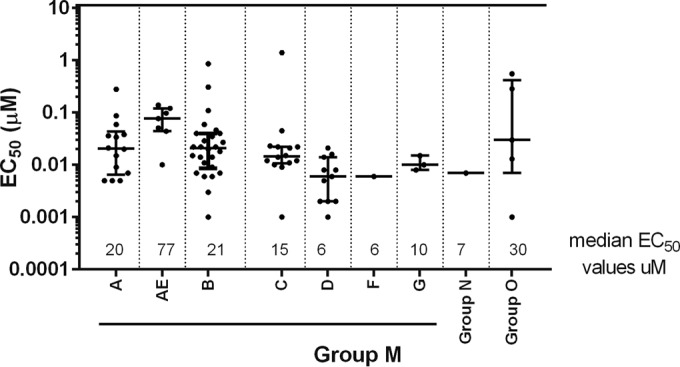
Anti-HIV-1 spectrum of BMS-955176 in PBMCs. Shown is the distribution of EC_50_s for clinical isolates evaluated in PBMCs; data are median values, with interquartiles. Each point is the mean of experiments performed in triplicate.

The spectrum of anti-HIV activity of BMS-955176 was also evaluated using a panel of HIV-1 clinical isolates assayed in PBMCs (medians with interquartiles are shown in Fig. 7; see also Table S4 in the supplemental material). A total of 82 isolates were tested from each of the 3 major groups: group M, including subtypes A (*n* = 14), B (*n* = 26), C (*n* = 14), D (*n* = 11), CRF01_AE (*n* = 7), F (*n* = 1), and G (*n* = 3), and groups N (*n* = 1) and O (*n* = 5). For 22 subtype B clinical isolates representing 96% of subtype B genetic variation in the region encompassing Gag 360 to 380, the meanBMS-955176 EC_50_ was 24 ± 24 nM, with a median of 17 nM. Within the subtype B group (*n* = 26), three less susceptible variants (mean EC_50_ of 123 ± 162 nM) contained the low-frequency SP1 variation Gag V370A/ΔT371 (1.9% in the LANL database). A fourth subtype B virus was insensitive (EC_50_ of 860 nM) and contained a low-frequency polymorphic triad (V362I/S373L/I376V; <0.5% in the 2012 LANL database). Thirteen out of 14 subtype C clinical isolates were inhibited, with a mean EC_50_ of 17 ± 11 nM and a median of 14 nM. One subtype C clinical isolate was insensitive to BMS-955176 (EC_50_ of 1,400 nM). Thirteen out of 14 subtype A viruses had a mean EC_50_ of 26 ± 25 nM, with a median of 20 nM. One subtype A isolate was 10.7-fold less sensitive (EC_50_ = 280 nM) than the rest of this cohort. The genetic correlates for reduced susceptibility in the subtype A and C viruses are not known. BMS-955176 was potent against subtypes D (*n* = 11), G (*n* = 3), and F (*n* = 1) and group N (*n* = 1), with mean EC_50_s of 7 ± 7, 11 ± 4, 5.9, and 7.2 nM, respectively. Activity was variable against group O viruses (*n* = 5), with an EC_50_ range of 1.3 to 550 nM and a median EC_50_ of 30 nM. A similar diversity was observed for subtype CRF01_AE viruses (*n* = 7), for which the EC_50_ range was 10 to 139 nM and the median EC_50_ was 30 nM. In conclusion, BMS-955176 exhibits good overall coverage of group M clinical isolates of subtypes A, CRF01_AE, B, C, D, G, and F. A single group N virus was sensitive, while the group O viruses were variable. The potency distribution is depicted in Fig. 7.

### BMS-955176 displays activity toward a broad panel of subtype B, C, and CRF_01_AE *gag/pr* recombinants in a single-cycle assay.

Given the PBMC data on clinical isolates suggesting modestly lower activity toward subtype CRF01_AE viruses, this subtype was more extensively studied by analyzing a panel of *gag/pr* CRF01_AE recombinants. However, because subtype CRF01_AE *gag/pr* recombinant viruses were compromised in fitness and did not grow well in a multiple-cycle assay in MT-2 cells, we used a modification of a published single-cycle assay method (46, 67, 68) to characterize this subtype's susceptibility to BMS-955176, comparing it to cohorts of subtype B and C recombinants. The modification involved production (in 293T cells) of MuLV *env*-pseudotyped viruses containing the *gag/pr* genes of the clinical isolates and then assaying for activity of infectious virus produced by that transfection by infection of fresh 293T cells. Data for each cohort (subtype B, *n* = 13; subtype C, *n* = 32; subtype CRF01_AE, *n* = 16) are shown in Fig. S7 in the supplemental material, with a statistical analysis in Table 4. Median FC-EC_50_s for subtypes B (1.1-fold) and C (3.4-fold) in this single-cycle assay (FC-EC_50_s from normalization to NLRepRlucP373S) were similar to results obtained using the multiple-cycle assay (Fig. 6B) (1.1- and 3.5-fold, respectively). The median FC-EC_50_ for CRF01_AE FC-EC50 was 2.4-fold (Table 4). Compared to median values, mean FC-EC_50_ for subtypes C and CRF01_AE were slightly higher, at 4.2- and 3.5-fold, respectively. The NFV control was equally active toward all subtypes, as expected (Table 4; see also Fig. S7 in the supplemental material). Overall, these data indicate that the activities of BMS-955176 toward subtype C and CRF01_AE viruses are similar to one another, and on average the mean FC-EC_50_ for these two subtypes are approximately 3-fold reduced relative to that for subtype B. As noted, the subtype B and C FC-EC_50_s are in alignment with those using the multiple-cycle antiviral luciferase reporter assay (Fig. 6B).

**TABLE 4:** Evaluation of Gag/Pr libraries derived from subtype B, C, and CRF01_AE clinical isolates in a single-cycle assay^*a*^

Subtype	*n*	BMS-955176				NFV			
		FC- EC_50_, mean	SD	FC- EC_50_, median	FC- EC_50_, median range	FC- EC_50_, mean	SD	FC- EC_50_, median	FC- EC_50_, median range
B	21	1.4	1.4	1.1	0.2–6.9	1.4	0.94	1.3	0.1–3.7
C	32	4.2	2.3	3.4	1.0–10.9	1.4	0.73	1.2	0.19–4.0
CRF_01AE	16	3.5	4.1	2.4	1.2–18.6	1	0.63	1.1	0.27–2.6

a FC relative to EC_50_ for WT (NLRepRlucP373S). Underlying values used for calculation of means and medians from experiments performed in triplicate. Coefficients of variation for all experiments were ≤200%.

### BMS-955176 is not cross-resistant to other classes of ARVs.

The antiviral (ARV) spectrum study of BMS-955176 was further validated with HIV-1 variants carrying substitutions known to encode ARV resistance. The mutations reported here include those with resistance to certain NRTIs, NNRTIs, PIs, and INSTIs). As expected, those viruses were resistant to the drugs within the same mechanistic class, with increased EC_50_s compared to the respective WT strain (Table 5). They remained sensitive to BMS-955176, indicating that there is no cross-resistance of BMS-955176 with other HIV-1 drug classes.

**TABLE 5:** Evaluation of BMS-955176 toward viruses resistant to other classes of antiretrovirals^*a*^

ARV	Class	Fold change in EC_50_ (SD) relative to parental strain					
		NRTI (184V)	NRTI (41L, 67N, 215F, 219E, 69N)^*b*^	NNRTI (103N, 181C)	PI (46I, 82F, 84V, 90 M)^*c*^	PI (54V, 82A, 84V 84V)^*d*^	INI (140S, 148H)
BMS-955176	MI	1.1 (0.29)	1.88 (0.21)	1.0 (0.20)	0.94 (0.24)	1.4	0.66 (0.18)
Lamivudine	NRTI	**>60 (59)**	**6.8 (3.9)**	1.07 (0.24)	1.28 (0.02)	ND	1.4 (0.11)
Zidovudine	NRTI	0.78 (0.03)	**79 (5)**	1.03 (0.17)	1.56 (0.46)	ND	0.82 (0.41)
Nevirapine	NNRTI	1.3 (0.2)	1.6 (0.2)	**>131 (108)**	0.83 (0.08)	ND	0.89 (0.59)
Efavirenz	NNRTI	1.0 (0.1)	ND	**55 (2.2)**	0.20 (0.01)	1.4	0.60 (0.3)
Rilpivirine	NNRTI	2.0 (0.3)	ND	**8.1 (1.5)**	2.6 (0.8)	ND	1.0 (0.5)
Darunavir	PI	1.2 (0.4)	ND	0.90 (0.1)	**41 (1.9)**	2.3	1.1 (0.1)
Atazanavir	PI	1.1 (0.1)	2.27 (0.02)	0.93 (0.03)	**38**^*c*^	**39**	1.0 (0.21)
**Raltegravir**	**INI**	1.9 (0.6)	ND	1.8 (0.4)	0.18 (0.07)	ND	**>340 (72)**

a Values are means (SD) of experiments performed a minimum of 3 times. ND, not determined. Boldface indicates fold change in EC_50_ > 6.8.

b AZT-resistant virus 7324-1.

c See the work of Gong et al. (41).

d *gag/pr* gene from PI-resistant virus (provided by Monique Nijhuis), transferred to an NLRepRluc backbone.

An absence of cross-resistance was also displayed by Gag polymorphic variants remaining sensitive to approved ARVs, such as raltegravir (INSTI), lamivudine (NRTI), tenofovir (NRTI), nevirapine (NNRTI), darunavir (PI), and atazanavir (PI), with EC_50_s comparable to those for the WT (Table 6).

**TABLE 6:** Antiviral activities of selected approved HIV-1 drugs toward Gag polymorphs^*a*^

Drug	Class	EC_50_ (SD), μM		
		Wild-type virus	Δ370 virus^*b*^	V370M virus^*c*^
BMS-955176	MI	0.0019 (0.0018)	0.013 (0.011)	0.010 (0.004)
Raltegravir	INI	0.0035 (0.0019)	0.0034 (0.0017)	0.0033 (0.00072)
Lamivudine	NRTI	0.39 (0.28)	0.47 (0.14)	0.36 (0.13)
Tenofovir	NRTI	0.0018 (0.00050)	0.0072 (0.0052)	0.00500 (0.0009)
Nevirapine	NNRTI	0.052 (0.043)	0.17 (0.062)	0.93 (0.029)
Darunavir	PI	0.0015 (0.00091)	0.0045 (0.0003)	0.0023 (0.00071)
Atazanavir	PI	0.0019 (0.00095)	0.0038 (0.0010)	0.0023 (0.0011)

a Values are from a multiple-cycle assay in MT-2 cells, with experiments performed a minimum of 3 times. The WT was NLRepRlucP373S.

b The SDM was NLRepRlucP373S/Δ370.

c Recombinant virus with *gag/pr* genes from a clinical isolate.

### BMS-955176 demonstrates no antagonistic interactions with antiretroviral drugs from different classes.

Potent activity, low human serum binding, and an absence of cross-resistance with other ARV classes suggest the potential for BMS-955176 to be a component of drug regimens, necessitating an understanding of the interactions between the different inhibitory mechanisms. Consequently, two-drug combination studies were performed with representative antiretrovirals from each of these classes: NRTIs (abacavir, lamivudine, emtricitabine, and tenofovir), NNRTIs (efavirenz and rilpivirine), PIs (ATV, DRV, and RTV), and INSTIs (integrase strand transfer inhibitors) (elvitegravir, raltegravir, and dolutegravir). The combination indices and asymptotic confidence intervals, which represent a measure of the variability in the data, for selected combinations are shown in Table S5 of the supplemental material. Additive or additive to synergistic effects were observed for all combinations tested. No antagonistic effects were observed for any of the combinations tested. In addition, no cytotoxicity was observed at the highest concentrations used in any of the combination assays.

## DISCUSSION

Infection with HIV-1 continues to be a serious health threat and one of the primary causes of death around the world. Despite advances in HIV treatment (3), there is a continuing need for the development of new antiretroviral drugs and regimens because of safety and long-term tolerability concerns with existing treatment options (7) and the emergence of resistance (6, 69).

MIs bind near a key structural element within the group-specific antigen (Gag polyprotein). This binding blocks the last protease cleavage event between Gag protein segments HIV-1 capsid (CA) protein p24 (24 kDa) and spacer peptide 1 (SP1), resulting in the release of immature noninfectious virus particles (9, 10, 15, 65). The clinical data for a first-generation MI, BVM, indicated that inhibition of maturation, *per se*, provided clinical benefit, but the failure of BVM in the clinic was a result of this compound not providing coverage of polymorphic variants found in approximately 50% of the general HIV-1 population. Thus, to succeed as a drug, an MI must be effective against isolates containing the polymorphic variations found in and nearby HIV-1 Gag SP1.

The strategy for the discovery and characterization of a second-generation MI with broadened antiviral coverage and reduced protein binding initially entailed screening using a cohort of site-directed substitutions representing the natural polymorphisms observed at positions 362 and 369 to 371 in primary screening. Analogs with broader polymorphic coverage were identified and optimized for potency, reduced serum binding, and appropriate pharmacokinetic properties so as to predict for QD dosing in humans. Mechanism-of-action studies were conducted to ensure that inhibition of maturation was maintained as the inhibitory mechanism. Key candidates were then further characterized by their activities toward libraries of *gag/pr* recombinant viruses derived from replacement of the *gag/pr* genes from clinical isolates into a laboratory backbone virus. This process led iteratively to the identification of BMS-955176, which contains major structure-activity-directed modifications to elements peripheral to the betulinic acid core structure, as well as to the core itself, compared to BVM. Key polymorphisms used to drive the structure-activity relationship (SAR) were V362I, V370A/M, ΔV370, and ΔV371, accounting for the ∼50% of viruses present in the HIV LANL sequence database (and hence the general population) which did not respond to BVM therapy. Subsequent antiviral coverage was evaluated against clinical isolates in PBMCs, while potential cross-resistance was monitored with a panel of viruses resistant to existing antiviral classes.

The initial site-directed mutant-mediated SAR identified BMS-1, which has an antiviral profile similar to that of BVM but a much reduced serum binding (54). The SAR progression led to candidates with greater polymorphic virus potencies. To ensure that candidates identified by primary screening were active toward a broad range of Gag diversity, several were evaluated against the library of *gag/pr* recombinant viruses. In each case, the spectrum of activity implied by the SDM-directed screening strategy was maintained in that broader analysis. *In vitro* analyses of a large database (*n* = 87) of clinical isolate *gag/pr* genes indicates that BMS-955176 exhibits potent antiviral activity against a cohort representing ∼96.5% of subtype B isolates. Using a fully infectious multiple-cycle assay, the mean EC_50_ of this subtype B cohort was 3.9 ± 3.4 nM, with a median value of 2.8 nM and a range of 0.7 to 22 nM. A similar analysis of 32 isolates of subtype C viruses found a mean EC_50_ of 10.9 ± 9.1 nM, a median value of 8.8 nM, and a range of 2.1 to 45 nM. Thus, BMS-955176 exhibits a clear improvement in potency against a number of polymorphic variants compared to BVM. BMS-955176 owes its improved potency at least in part to higher-affinity binding to its HIV-1 Gag target in these polymorphism-containing viruses, which compensates for their more rapid cleavage by HIV-1 protease (33). A mechanistic analysis of the interplay between MI Gag affinity and Gag polymorphic cleavage rates was also performed (Z. Lin, J. Cantone, H. Lu, B. Nowicka-Sans, T. Protack, T. Yuan, H. Yang, Z. Liu, D. Drexler, A. Regueiro-Ren, N. A. Meanwell, M. Cockett, M. Krystal, M. Lataillade, and I. B. Dicker, unpublished data).

When evaluated against clinical isolates in PBMCs, BMS-955176 exhibited a mean EC_50_ of 24 ± 24 nM against a cohort (*n* = 22) of subtype B viruses. Activity was also observed against viruses from subtypes A, C, D, F, and G, with EC_50_s between 5.9 nM and 87 nM (*n* = 41/43 isolates). Compared to subtype B, clinical isolates from the CRF01_AE subtype were approximately 2- to 3-fold less susceptible to BMS-955176 in PBMC-based antiviral assays. This was explored further using *gag/pr* recombinant CRF01_AE viruses. Though these recombinants did not grow well enough to be studied in a multiple-cycle format, their susceptibility to BMS-955176 was compared to those of subtype B and C cohorts in a single-cycle MuLV pseudotype assay. CRF01_AE susceptibility to BMS-955176 was similar to that of subtype C viruses (both have median FC-EC_50_s approximately 3-fold reduced versus the median of subtype B viruses). In addition, ongoing preclinical work and clinical studies are under way to help define the genotypic correlates of resistance in different subtypes.

In cell culture, the range of values for the concentration producing a 50% effect of BMS-955176 against 7 common CXCR4-utilizing laboratory strains of HIV-1 was 0.9 to 11 nM. The CCR5-utilizing viruses BAL and JRFL were susceptible to BMS955176, with EC_50_s of 0.7 and 2.5 nM, respectively. One of three HIV-2 strains tested was susceptible, with indications that susceptibility maps to the CA/SP1 region.

BMS-955176 exhibits a modest (5.4-fold) reduction in antiviral activity in the presence of 40% human serum supplemented with additional HSA to match physiological levels (total = 45 mg/ml), giving an EC_90_ of 14 nM against the WT screening virus under this condition. Protein binding in 100% human serum was 86% using an ultracentrifugation method, similar to the value implied by the 5.4-fold reduction in cell culture (82%). Consequently, both the cell culture- and *ex vivo*-derived values indicate an acceptable free fraction of BMS-955176 in serum (18% versus 14%, respectively). Cytotoxicity (CC_50_) values in 5 cell lines ranged from 2.3 to >15 μM; a high therapeutic index, ∼4,500, was determined for BMS-955176 in MT-2 cells. The significance of *in vitro* cytotoxicity is unclear, as BMS-955176 has been safe and well tolerated in phase 2a clinical studies (32).

Mechanism-of-action studies prove that BMS-955176 is a true MI, with an MOA distinct from that of the current ARVs. BMS-955176 inhibits late in the HIV-1 life cycle, specifically inhibiting HIV-1 protease cleavage at the CA (p24)/SP1 junction in the context of HIV-1-infected cells and in fully assembled HIV-1 Gag VLPs *in vitro*. [^3^H]BMS-955176 binds specifically, saturably, and reversibly to purified HIV-1 Gag VLPs, and its binding is dose dependently inhibited by BVM and related MIs. In addition, a recent LC/MS analysis of peptides produced from HIV-1 protease cleavage of HIV-1 VLPs *in vitro* found that BMS-955176 more efficiently inhibits p25 cleavage than does BVM (33; Li et al., unpublished). Altogether, the cell and biochemical data indicate that BMS-955176 inhibits late in the HIV-1 life cycle by specific binding to immature CA structures at or near the CA (p24)/SP1 junction, thereby inhibiting cleavage at that site.

As expected for an agent with a different MOA, BMS-955176 retains complete activity against reverse transcriptase, protease, and integrase inhibitor-resistant viruses, with EC_50_s similar to those for wild-type viruses. Conversely, the potency of currently approved nucleotide/nucleoside reverse transcriptase inhibitors (NRTIs), nonnucleoside reverse transcriptase inhibitors (NNRTIs), protease inhibitors (PIs), and INIs was undiminished when tested against viruses with reduced susceptibility to BMS-955176. No antagonism was observed between BMS-955176 and representative NRTI, NNRTI, PI, and INI ARVs in two-drug combination studies, with all combinations producing at least additive effects. The lack of cross-resistance and antagonism indicates that BMS-955176 should be amenable for use in combination with agents of any of these drug classes.

Altogether, these data highlight the potential for BMS-955176 to exert a strong clinical response, and such an improved response has been realized in a 10-day phase 2a monotherapy study, with subtype B patients, regardless of Gag genotype, responding similarly (32; C. S. D. Hwang, C. Sobotha, M. Boffito, H. Sevinsky, N. Ray, P. Ravindran, H. Xiao, M. Krystal, I. Dicker, D. Grasela, and M. Lataillade, unpublished data). This clinical result highlights the successful application of our preclinical MI discovery strategy, with the clinical data supporting the continued development of BMS-955176 in phase 2b trials.

## Supplementary Material

Supplemental material
