# Supplementary material for: Identification and Characterization of BMS-955176, a Second-Generation HIV-1 Maturation Inhibitor with Improved Potency, Antiviral Spectrum, and Gag Polymorphic Coverage
Source: Antimicrob Agents Chemother. 2016 Jun 20;60(7):3956–69. doi: 10.1128/AAC.02560-15 (PMC4914680; doi:10.1128/AAC.02560-15)
Supplement: Supplemental material [file supp_60_7_3956__index.html]

Identification and Characterization of BMS-955176, a Second-Generation HIV-1 Maturation Inhibitor with Improved Potency, Antiviral Spectrum, and Gag Polymorphic Coverage — Supplemental material 

# Identification and Characterization of BMS-955176, a Second-Generation HIV-1 Maturation Inhibitor with Improved Potency, Antiviral Spectrum, and Gag Polymorphic Coverage

## Supplemental material

- Supplemental file 1 -

  Additional experimental details and supplemental tables and figures.

  PDF, 361K
