# Supplementary material for: Identification and Characterization of BMS-955176, a Second-Generation HIV-1 Maturation Inhibitor with Improved Potency, Antiviral Spectrum, and Gag Polymorphic Coverage
Source: Antimicrob Agents Chemother. 2016 Jun 20;60(7):3956–69. doi: 10.1128/AAC.02560-15 (PMC4914680; doi:10.1128/AAC.02560-15)
Supplement: Supplemental material [file AAC.02560-15_zac007165282so1.pdf]

Supplementary Experimental

Cytotoxicity assays. CC_{50} values (concentrations of drug required to reduce cell viability by 50%) of BMS-955176 were determined in MT-2 (human T cell line), Huh7 (human hepatocarcinoma), C33a (human cervical carcinoma), HeLa (epithelial cells) and MDCK (Madin-Darby canine kidney epithelial cells) after a 4 - 6 day incubation in the presence of serially-diluted compound. Cell viability was determined using a respiration assay (MTS, Promega) according to the manufacturer's protocol.(1) CC_{50}s values were calculated by using the exponential form of the median effect equation, where percent inhibition = 1/[1+ (CC_{50}/drug conc.)m], where m is a parameter that reflects the slope of the concentration-response curve. CC_{50} values (cell line) were 9.2 +/- 4 µM (MT-2, human T cell line), 3.8 µM, (Huh7, human hepatocarcinoma), >15 µM (C33a, human cervical carcinoma), 2.3 µM, (HeLa, epithelial cells) and 3.9 µM, (MDCK, Madin-Darby canine kidney epithelial cells). The CC_{50}s in the five cell lines are suggestive of moderate cytotoxicity, while the therapeutic index in MT-2 cells is relatively high (4842), indicating that the effects of BMS-955176 are virus-specific. In addition, in initial clinical studies, BMS-955176 has been safe and well tolerated.(2-4)

Off-target activity assays. BMS-955176 was examined in an *in vitro* HIV-1 reverse transcriptase assay (EnzChek® Reverse Transcriptase Assay Kit, Thermo Fisher, Waltham, MA, USA), an influenza virus polymerase assay,(5) an HIV-1 integrase inhibitor binding assay(6) and an HIV-1 protease cleavage assay (SensoLyte® 520 HIV-1 Protease Fluorimetric Assay, AnaSpec, San Jose, CA).

Two-drug combination assays. MT-2 cells were infected with NLRepRlucP373S virus at an MOI of 0.005, and the cells were seeded at a density of 0.1×10^6 cells/mL onto 96-well plates containing 2-3 fold serially diluted compounds. The drugs were diluted in a fixed and multiple molar ratios, representing 1 : 1, 1 : 2.5, and 2.5 : 1 times the known EC_{50} for each drug (determined in prior experiments). The drug serial dilutions spanned a range of concentrations near the EC_{50} value of each compound, so that equivalent antiviral activities could be compared. After a 4 - 5 day incubation at

$37°C/CO_2$, virus yields were determined by the *Renilla* luciferase activity (Dual-Luciferase® Reporter Assay System, Promega). There was no cytotoxicity associated with any of the drug combinations (no reduction in cell numbers by a companion assay of cells alone exposed to the same drug combinations). To determine antiviral effects of different drug combinations, combination indices (CIs) were calculated according to the method of Chou.(7) Concentration-response curves were estimated for each individual drug and every combination using the median-effect equation, $Fa = 1/[1+ (ED_{50}/\text{drug concentration})^m]$, where Fa stands for fraction affected.(8). The equation was fit using a nonlinear regression routine (Proc Nlin) in PC SAS version 8.01 (SAS Institute Inc., SAS Version 8.01, Cary, NC: SAS Institute Inc., 1990). Curves with an R-square values less than 0.90 were deleted.

HIV-1 cell fusion assay.

The ability to inhibit cell fusion events mediated by the HIV-1 gp160 envelope protein was analyzed (events up to and including fusion) by a luciferase readout as described,(9, 10) using 1) HeLa C14 cells (CD4/CXCR4/CCR5-positive) target cells which express TRE (tet-responsive element) controlled firefly luciferase reporter gene, and 2) NL-TOE donor cells which express HIV-1 envelope and tTA protein that trans-activates TRE-controlled gene expression upon cell-cell fusion.

Serum binding assay. Serum binding was determined using an ultracentrifugation method.(11) First, 53 µM [^{3}H]-BMS-955176 (specific activity 18.9 Ci/mmol) was diluted 10-fold in 100% DMSO, and 10-µl aliquots were mixed with 1 mL of human serum in a 1.5 mL Eppendorf tube (Beckman Coulter, Brea, CA, USA). After 1-hour incubation at 37°C, the samples were centrifuged for 18 hours at 37°C in a TLA100.3 rotor in an Optima TLX ultracentrifuge at 58,000 rpm. One hundred µL fractions were collected from the top of the tubes. The fraction with < 1% of the total starting protein concentration (2nd fraction from the top out of 10 fractions) was taken as the protein-free fraction, and was measured for [^{3}H]-BMS-955176-associated radioactivity. That value was compared to the total radioactivity used in the assay.

Supplementary materials, tables and figures

Supplementary Table S1. Differentiation of BMS-955176 from Bevirimat toward Gag Polymorphic Viruses

	**BMS-955176**			**Bevirimat**			**Nelfinavir**		
	EC_{50}, μM	Fold (*vs.* WT)	N	EC_{50}, μM	Fold (*vs.* WT)	N	EC_{50}, μM	Fold (*vs.* WT)	N
Wild-type	0.0019	-	42	0.010	-	56	0.004	-	120
V362I	0.0045	2.2	7	0.074	7.4	4	0.005	1.3	14
V370A	0.0027	1.4	42	0.55	55	30	0.004	1.2	97
V370M	0.0028	1.5	2	0.18	181	1	0.005	1.4	2
ΔV370	0.013	6.8	41	≥10	≥ 1000	16	0.005	1.3	92

Amino acid changes were introduced into NLRepRlucP373S (WT virus) and evaluated for antiviral activity in MT2 cells by luciferase activity as described in Materials and Methods. N = number of experiments performed, with each run performed in triplicate.

Supplementary Table S2. Human Serum Effect on Antiviral Activity of BMS-955176

	EC_{50} ± SD (nM)	N	FC *vs.* 10% FBS
10% FBS	1.9 ± 1.8	226	1
10% FBS + 40% HS + 27 mg/mL HSA	10.2 ± 6.0	76	5.4
10% FBS + 45 mg/mL HSA	4.7	2	2.5
10% FBS + 40% HS	4.9	1	2.6
10% FBS + 1 mg/mL AGP	1.0	1	0.5
10% FBS + 45 mg/mL HSA + 1 mg/mL AGP	6.2	1	3.3

SD standard deviation, FC=FC-EC50

Supplementary Table S3. Gag Cleavage Site Sequences in HIV-2 Strains Evaluated for BMS-955176 Susceptibility

Type														p17	p24																											
		120	121	122	123	124	125	126	127	128	129	130	131	132	133	134	135	136	137	138	139	140	141	142	143	144	145	146	147	148	149	150	151	152	153	154	155	156	157	158	159	160	161
HIV-1	HXB2	A	D	T	G	H	S	N	Q	V	S	Q	N	Y	P	I	V	Q	N	I	Q	G	Q	M	V	H	Q	A	I	S	P	R	T	L	N	A	W	V	K	V	V	E	E
HIV-1	Lab	.	.	.	.	N	N	S	.	.	.	.	.	.	.	.	.	.	.	L	.	.	.	.	.	.	.	.	.	.	.	.	.	.	.	.	.	.	.	.	.	.	.
HIV-1	subbtype B Cons	.	.	.	.	N	.	S	.	.	.	.	.	.	.	.	.	.	.	L	.	.	.	.	.	.	.	.	.	.	.	.	.	.	.	.	.	.	.	.	.	.	.
HIV-1	subbtype C Cons	.	A	D	.	—	—	—	K	.	.	.	.	.	.	.	.	.	.	L	.	.	.	.	.	.	.	.	.	.	.	.	.	.	.	.	.	.	.	.	I	.	.
HIV-2	ROD	R	P	.	A	P	[.]	S	[E]	K	G	G	.	[.]	.	—	.	.	[H]	V	G	.	N	Y	T	.	[I]	P	L	.	.	.	.	.	.	.	.	.	.	L	.	.	.
HIV-2	CBL-20	R	P	.	A	P	P	S	G	R	G	G	.	F	.	—	.	.	Q	A	G	.	N	Y	.	.	V	P	L	.	.	.	.	.	.	.	.	.	.	L	.	.	.
HIV-2	CBL-23	R	P	.	A	P	P	S	G	K	G	G	.	F	.	—	.	.	Q	V	G	.	N	Y	T	.	V	P	L	.	.	.	.	.	.	.	.	.	.	L	.	.	.

Type														p24	SP1													SP1	NC														
		351	352	353	354	355	356	357	358	359	360	361	362	363	364	365	366	367	368	369	370	371	372	373	374	375	376	377	378	379	380	381	382	383	384	385	386	387	388	389	390	391	392
HIV-1	HXB2	Q	G	V	G	G	P	G	H	K	A	R	V	L	A	E	A	M	S	Q	V	T	N	S	A	T	I	M	M	Q	R	G	N	F	R	N	Q	R	K	I	V	K	C
HIV-1	Lab	.	.	.	.	.	.	.	.	.	.	.	.	.	.	.	.	.	.	.	.	.	.	P	.	.	.	.	I	.	K	.	.	.	.	.	.	.	.	T	.	.	.
HIV-1	subbtype B Cons	.	.	.	.	.	.	.	.	.	.	.	.	.	.	.	.	.	.	.	.	.	.	.	.	.	.	.	.	.	.	.	.	.	.	.	.	.	.	T	.	.	.
HIV-1	subbtype C Cons	.	.	.	.	.	.	.	.	.	.	.	.	.	.	.	.	.	.	.	A	N	.	T	—	N	.	.	.	.	.	S	.	.	K	G	P	K	R	.	.	.	.
HIV-2	ROD	.	.	.	.	.	.	.	Q	.	.	.	L	M	.	.	.	L	K	E	[.]	[I]	[G]	P	.	P	.	P	F	A	A	A	Q	Q	.	—	—	—	.	A	[F]	.	.
HIV-2	CBL-20	.	.	.	.	.	.	.	Q	.	.	.	L	M	.	.	.	L	K	E	A	L	A	P	P	P	.	P	F	A	A	A	Q	Q	.	—	—	—	.	A	I	.	.
HIV-2	CBL-23	.	.	.	.	.	.	.	Q	.	.	.	L	M	.	.	.	L	K	E	A	L	T	P	.	P	.	P	F	A	A	A	Q	Q	K	—	—	—	R	T	I	.	.

Type														NC	P1																	P1	P6										
		420	421	422	423	424	425	426	427	428	429	430	431	432	433	434	435	436	437	438	439	440	441	442	443	444	444	444	445	446	447	448	449	450	451	452	453	454	455	456	457	458	459
HIV-1	HXB2	G	H	Q	M	K	D	C	T	E	R	Q	A	N	F	L	G	K	I	W	P	S	Y	K	G	R	—	—	P	G	N	F	L	Q	S	R	P	E	P	T	A	P	P
HIV-1	Lab	.	.	.	.	.	.	.	.	.	.	.	.	.	.	.	.	.	.	.	.	.	H	.	.	.	.	.	.	.	.	.	.	.	.	.	.	.	.	.	.	.	.
HIV-1	subbtype B Cons	.	.	.	.	.	.	.	.	.	.	.	.	.	.	.	.	.	.	.	.	.	H	.	.	.	.	.	.	.	.	.	.	.	.	.	.	.	.	.	.	.	.
HIV-1	subbtype C Cons	.	.	.	.	.	.	.	.	.	.	.	.	.	.	.	.	.	.	.	.	.	H	.	.	.	.	.	.	.	.	.	.	.	.	.	.	.	.	.	.	.	.
HIV-2	ROD	.	.	[I]	.	[T]	N	.	P	D	.	.	.	G	.	.	.	[L]	G	P	W	G	K	.	P	.	N	F	.	V	A	Q	V	P	Q	G	L	T	.	.	.	.	.
HIV-2	CBL-20	.	.	V	.	A	N	.	P	D	.	.	.	G	.	.	.	M	G	P	W	G	K	.	P	.	N	F	.	M	A	Q	A	P	Q	G	L	I	.	.	.	.	.
HIV-2	CBL-23	.	.	V	I	A	N	.	P	D	.	.	V	G	.	.	.	M	G	P	R	G	K	.	P	.	N	F	.	V	A	Q	V	P	Q	G	L	T	.	.	.	.	.

key: Boxed amino acid in HIV2-ROD differs from HIV-2 CBL-20 & CBL-23. HIV-1 and HIV-2 *gag* segments (p17, p24, p2, NC, p1 & p6) as defined by Los Alamos National Lab published sequences

Supplementary Table S4. Activity of BMS-955176 against Clinical Isolates

Group	Predominant SP1 QVT[a,] Genotypes	# Isolates	BMS-955176 Mean EC_{50} (nM) ± SD	Range EC_{50}, (nM)	Median EC_{50}, (nM)
M	B, (all)	26	67 ± 172	1-858	21
	B (96% of Gag diversity)	22	24 ± 24	1-110	17
	A, V370A/Δ371	14	44 ± 72	5-280	21
	A[b]	13	26 ± 25	5-87	20
	AE, V370A/Δ371	7	77 ± 46	10-139	77
	C, ΔV370, V370A/ΔT371	14	116 ± 360	1-1400	14
	C	13	17 ± 11	1-45	14
	C[b]	1	1400	1400	1400
	D, (V370A)	11	7 ± 7	1-21	6
	G, (V370A)	3	11 ± 4	8-15	10
	F, (V370A)	1	5.9	6	6
N		1	7.2	7	7
O		5	170 ± 240	1-552	30

[a]Gag amino acids Q369, V370, T371; Los Alamos National Laboratories database as of 2012; [b]subtype A and C isolates with reduced BMS-955176 susceptibility

Supplementary Table S5. The combination indices, and overall interaction results, for BMS-955176 in combination with approved and preclinical antiretroviral compounds from different inhibitory classes

Molar Ratio (EC50 Ratio)[a]	Combination Indices at % HIV Inhibition[b] (Confidence Interval)			
	50%	75%	90%	Overall Result
Nucleoside Reverse transcriptase inhibitors				
BMS-986001 - Expt 1				
3:400 (1:1)	0.86 (0.80, 0.93)	0.85 (0.76, 0.94)	0.88 (0.74, 1.03)	
3:1000 (1:2.5)	0.85 (0.78, 0.92)	0.86 (0.76, 0.96)	0.92 (0.75, 1.08)	Additive
3:160 (2.5:1)	0.80 (0.75, 0.86)	0.75 (0.68, 0.82)	0.72 (0.62, 0.83)	
BMS-986001- Expt 2				
3:400 (1:1)	0.84 (0.79, 0.89)	0.89 (0.82, 0.96)	0.98 (0.86, 1.10)	
3:1000 (1:2.5)	0.96 (0.91, 1.02)	0.96 (0.88, 1.03)	0.99 (0.87, 1.11)	Additive
3:160 (2.5:1)	0.94 (0.89, 0.98)	0.95 (0.88, 1.01)	0.98 (0.87, 1.08)	
Abacavir- Expt 1				
3:400 (1:1)	0.84(0.78,0.90)	0.90(0.81,0.98)	1.01(0.86,1.16)	
3:1000 (1:2.5)	0.98(0.92,1.05)	1.00(0.90,1.10)	1.05(0.89,1.21)	Additive
3:160 (2.5:1)	0.86(0.81,0.91)	0.84(0.77,0.90)	0.85(0.74,0.96)	
Abacavir-Expt 2				
3:400 (1:1)	0.83(0.79,0.88)	0.81(0.75,0.88)	0.83(0.73,0.93)	Additive
3:1000 (1:2.5)	0.87(0.82,0.92)	0.85(0.78,0.92)	0.85(0.74,0.96)	

Supplementary Table S5. The combination indices, and overall interaction results, for BMS-955176 in combination with approved and preclinical antiretroviral compounds from different inhibitory classes

3:160 (2.5:1)	0.77(0.73,0.81)	0.83(0.76,0.89)	0.92(0.81,1.04)	
		Non-Nucleoside Reverse Transcriptase Inhibitors		
Rilpivirine- Expt 1				
15:2 (1:1)	1.07(1.00,1.15)	1.00(0.91,1.10)	0.94(0.79,1.08)	
3:1 (1:2.5)	0.95(0.89,1.02)	0.88(0.80,0.97)	0.82(0.70,0.94)	Additive
75:4 (2.5:1)	0.86(0.79,0.92)	0.84(0.76,0.93)	0.83(0.69,0.96)	
Rilpivirine- Expt 2				
15:2 (1:1)	0.98(0.91,1.05)	1.02(0.92,1.11)	1.05(0.89,1.21)	
3:1 (1:2.5)	0.94(0.88,1.01)	1.03(0.93,1.14)	1.13(0.95,1.31)	Additive
75:4 (2.5:1)	0.67(0.61,0.73)	0.91(0.80,1.01)	1.23(0.99,1.46)	
		Integrase Inhibitors		
dolutegravir- Expt 1				
15:2 (1:1)	0.83(0.74,0.93)	0.89(0.75,1.02)	0.94(0.71,1.17)	
3:1 (1:2.5)	0.89(0.79,0.98)	0.87(0.74,0.99)	0.85(0.65,1.04)	Additive
75:4 (2.5:1)	0.91(0.83,0.99)	0.78(0.69,0.88)	0.68(0.54,0.81)	
dolutegravir- Expt 2				
15:2 (1:1)	0.90(0.81,0.99)	0.93(0.80,1.05)	0.97(0.76,1.18)	
3:1 (1:2.5)	0.81(0.73,0.89)	0.84(0.72,0.95)	0.87(0.68,1.07)	Additive
75:4 (2.5:1)	0.88(0.80,0.96)	0.89(0.78,1.01)	0.91(0.73,1.10)	

Supplementary Table S5. The combination indices, and overall interaction results, for BMS-955176 in combination with approved and preclinical antiretroviral compounds from different inhibitory classes

elvitegravir- Expt 1				
15:2 (1:1)	1.08(1.00,1.17)	1.05(0.94,1.15)	1.02(0.85,1.18)	
3:1 (1:2.5)	1.05(0.97,1.13)	0.99(0.88,1.09)	0.94(0.78,1.10)	Additive
75:4 (2.5:1)	0.98(0.90,1.05)	0.94(0.84,1.04)	0.91(0.77,1.06)	
elvitegravir-Expt 2				
15:2 (1:1)	0.90(0.83,0.96)	0.83(0.74,0.93)	0.79(0.65,0.93)	
3:1 (1:2.5)	0.92(0.85,1.00)	0.86(0.76,0.96)	0.81(0.66,0.96)	Additive
75:4 (2.5:1)	0.82(0.74,0.89)	0.95(0.83,1.08)	1.12(0.89,1.35)	
		atazanavir (PI)		
Atazanavir- Expt 1				
2:1 (1:1)	0.92(0.84,1.00)	1.06(0.93,1.18)	1.22(0.99,1.45)	
4:5 (1:2.5)	0.94(0.87,1.00)	0.91(0.82,1.00)	0.88(0.75,1.02)	Additive
5:1 (2.5:1)	0.79(0.73,0.85)	0.82(0.74,0.91)	0.85(0.71,0.99)	
Atazanavir- Expt 2				
2:1 (1:1)	0.99(0.92,1.06)	0.92(0.83,1.00)	0.85(0.72,0.97)	
4:5 (1:2.5)	1.01(0.93,1.08)	0.97(0.87,1.07)	0.94(0.79,1.09)	Additive
5:1 (2.5:1)	0.98(0.90,1.06)	1.02(0.91,1.14)	1.07(0.88,1.26)	

[a]Molar ratio of BMS-955176 to comparator compound.

[b]A lower bound of the asymptotic confidence interval greater than 1 indicates antagonism, an upper bound of less than 1 indicated synergism and a value of 1 being contained within the interval indicates additivity. The 95% confidence intervals are shown in parenthesis, and represent a measure of variability in the data.

Supplementary Figure S6. Western analysis of Gag cleavage ± BMS-955176

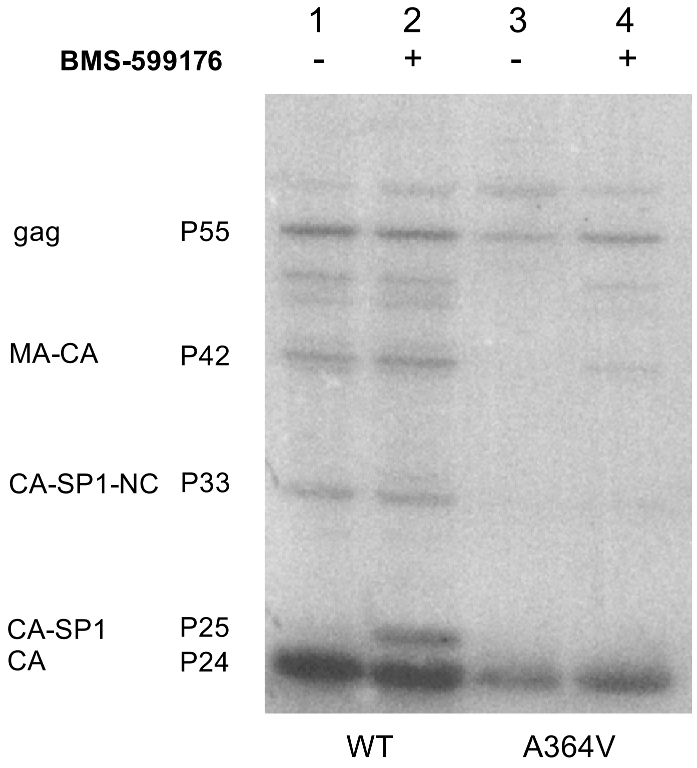

Inhibition of HIV-1 Gag cleavage of HIV-1 virus in cells; “-“ = no BMS-955176, “+” = 200 nM of BMS-955176. Western analysis using a primary anti-p24 monoclonal antibody (Perkin Elmer, NEA9306001), as described in Material and Methods.

Supplementary Figure S7. Analysis of *gag/pr* recombinant subtype B, C and CRF01_AE viruses using a single cycle assay

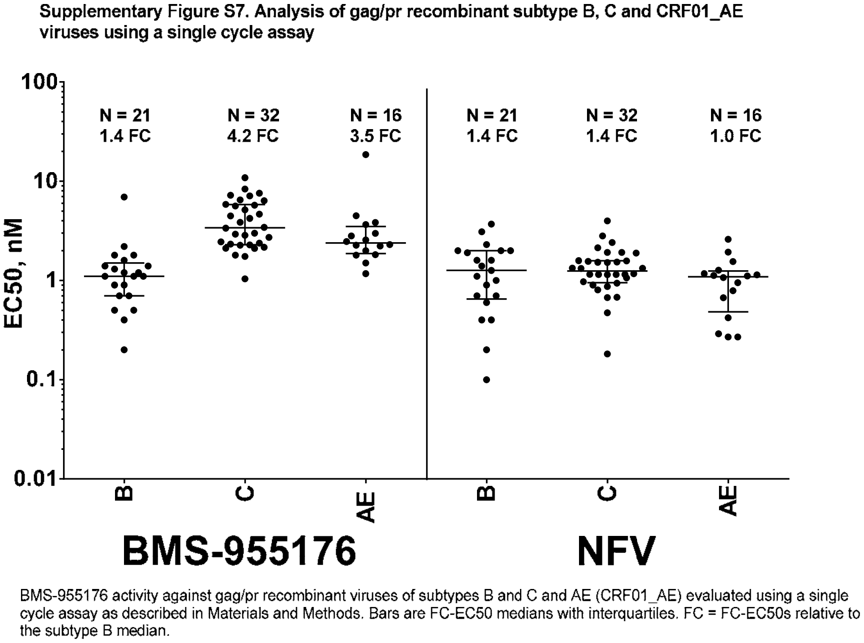

BMS-955176 activity against *gag/pr* recombinant viruses of subtypes B and C and AE (CRF01_AE) evaluated using a single cycle assay as described in Materials and Methods. Bars are FC-EC_{50} medians with interquartiles. FC = FC-EC_{50}s relative to the subtype B median. Values above datapoints are means, see Table 4 in manuscript for details.

1. **Tominaga H, Ishiyama M, Ohseto F, Sasamoto K, Hamamoto T, Suzuki K, Watanabe M.** 1999. A water-soluble tetrazolium salt useful for colorimetric cell viability assay. Analytical Communications **36:**47-50.
2. **Hwang C, Schürmann D, Sobotha C, Sevinsky H, Ravindran P, Xiao H, Ray N, Krystal M, Dicker I, Lataillade M.** BMS-955176: Antiviral Activity and Safety of a Second-Generation HIV-1 Maturation Inhibitor, p Abstract 114LB. *In* (ed),
3. **Hwang C, Schürmann D, Sobotha C, Sevinsky H, Ravindran P, Xiao H, Ray N, Krystal M, Dicker I, Grasela D, Lataillade M.** Second-Generation HIV-1 Maturation Inhibitor BMS-955176: Antiviral Activity and Safety with Atazanavir ± Ritonavir, p Abstract TUAB0106LB. *In* (ed),
4. **Hwang C, Schartman R, Sobotha C, Sevinsky H, Ray N, Ravindran P, Xiao H, Krystal M, Dicker I, Grasela D, Lataillade M.** Overall Antiviral Activity and Safety Results from the Phase IIa Proof-of-Concept Study (AI468002). , p Abstract PS10/15. *In* (ed),
5. **Gerritz SW, Cianci C, Kim S, Pearce BC, Deminie C, Discotto L, McAuliffe B, Minassian BF, Shi S, Zhu S, Zhai W, Pendri A, Li G, Poss MA, Edavettal S, McDonnell PA, Lewis HA, Maskos K, Mortl M, Kiefersauer R, Steinbacher S, Baldwin ET, Metzler W, Bryson J, Healy MD, Philip T, Zoeckler M, Schartman R, Sinz M, Leyva-Grado VH, Hoffmann HH, Langley DR, Meanwell NA, Krystal M.** 2011. Inhibition of influenza virus replication via small molecules that induce the formation of higher-order nucleoprotein oligomers. Proc Natl Acad Sci U S A **108:**15366-15371.
6. **Dicker IB, Terry B, Lin Z, Li Z, Bollini S, Samanta HK, Gali V, Walker MA, Krystal MR.** 2008. Biochemical analysis of HIV-1 integrase variants resistant to strand transfer inhibitors. J Biol Chem **283:**23599-23609.

7. **Chou T-C.** 1991. The median-effect principle and the combination index for quantitation of synergism and antagonism, p 61-101. *In* Chou T-C, Rideout DC (ed), Synergism and antagonism in chemotherapy. Academic Press, San Diego, CA.
8. **Anonymous.** 1993. ACTG Virology Manual for HIV laboratory Version 2.0. DAIDS-NIAID
9. **Zhou N, Nowicka-Sans B, Zhang S, Fan L, Fang J, Fang H, Gong YF, Eggers B, Langley DR, Wang T, Kadow J, Grasela D, Hanna GJ, Alexander L, Colonno R, Krystal M, Lin PF.** 2011. In vivo patterns of resistance to the HIV attachment inhibitor BMS-488043. Antimicrob Agents Chemother **55:**729-737.
10. **Guo Q, Ho HT, Dicker I, Fan L, Zhou N, Friborg J, Wang T, McAuliffe BV, Wang HG, Rose RE, Fang H, Scarnati HT, Langley DR, Meanwell NA, Abraham R, Colonno RJ, Lin PF.** 2003. Biochemical and genetic characterizations of a novel human immunodeficiency virus type 1 inhibitor that blocks gp120-CD4 interactions. J Virol **77:**10528-10536.
11. **Nakai D, Kumamoto K, Sakikawa C, Kosaka T, Tokui T.** 2004. Evaluation of the protein binding ratio of drugs by a micro-scale ultracentrifugation method. J Pharm Sci **93:**847-854.
